# Our New Volume

**Published:** 1882-01

**Authors:** 


### EDITORIAL DEPARTMENT.

OUR NEW VOLUME.—We take pleasure in calling the attention of our readers to the many improvements which we have introduced in the present number of the INDEPENDENT PRACTITIONER. Our handsome and tasty cover will doubtless receive the commendation of all, while the large list of eminent collaborators is a guarantee of the character of the contents of the volume just begun. While preserving the features that have hitherto made the INDEPENDENT PRACTITIONER a welcome monthly visitor, we have added a department of "*Clinical Reports*," the first of which appears in this number; a department of "*Original Translations and Abstracts*," which will be filled with condensations of the choicest and most practical contributions to foreign and domestic medical literature, and a department of "*Current News and Opinion*," in the form of brief and interesting paragraphs of happenings in the medical world.

The INDEPENDENT PRACTITIONER is no longer a venture, but an established and paying investment. We shall endeavor to make it better with each succeeding number, and shall not be satisfied until we are able to lay before our readers the best, cheapest and handsomest monthly medical journal published.

We shall do what in us lies to elevate the standard of professional education, and constantly strive to break down the artificial boundary lines between medicine and dentistry. In this endeavor we ask, and expect the hearty support of all who desire to see dentistry take its proper place as a scientific specialty of general medicine.

Five thousand copies of this number will be mailed to physicians and dentists in different parts of the country. We not only expect to retain all our old subscribers, but to add many new friends to our roll. We call attention to the exceptional advantages to be gained by subscribing to other journals, or purchasing medical works through us.

